# Data on the polymorphic sites in the chloroplast genomes of the sunflower alloplasmic CMS lines

**DOI:** 10.1016/j.dib.2019.104072

**Published:** 2019-05-26

**Authors:** Kirill Azarin, Maksim Makarenko, Alexander Usatov, Oleg Gorbachenko, Alexey Kovalevich, Vera Gavrilova

**Affiliations:** a Southern Federal University, Rostov-on-Don, Russia; b Zlhdanov Don Experiment Station, All Russia Research Institute of Oil Crops, pos. Oporny, Rostov region, Russia; c The N.I. Vavilov All Russian Institute of Plant Genetic Resources, Saint Petersburg, Russia

**Keywords:** Chloroplast genome, Polymorphic site, Alloplasmic CMS line, Sunflower

## Abstract

Data presents the chloroplast genome sequences of the five sunflower alloplasmic cytoplasmic male sterility (CMS) lines obtained with using the Illumina MiSeq, HiSeq and NextSeq platforms. The sunflower alloplasmic CMS lines has the same nuclear genome from line HA89, but they differ in cytoplasmic genomes, inherited from annual (PET1, PET2 - *H. petiolaris*, ANN2 - *H. annuus*) and perennial (MAX1 - *H. maximilliani*) species of the genus *Helianthus* L. The chloroplast genomes were annotated. Also presented is a dataset of variable sites such as single nucleotide polymorphism (SNP), simple sequence repeat (SSR), insertion and deletion (INDEL) in the chloroplast genome of the sequenced alloplasmic lines. The raw reads are available in FIGSHARE (https://doi.org/10.6084/m9.figshare.7520183). The complete chloroplast genome sequences for the sunflower alloplasmic lines are available in GenBank NCBI under the accessions MK341448.1-MK341452.1; the remaining data are provided with this article.

**Table:** Specifications Table

Subject area	*Biology*
More specific subject area	*Genomics of crop plants*
Type of data	*Tables and figures, Deoxyribonucleic acid (DNA) sequence*
How data was acquired	*Illumina MiSeq; Illumina NextSeq 500; Illumina HiSeq 2000*
Data format	*Raw and analyzed*
Experimental factors	*De novo assemblies were created using SPAdes Genome Assembler v 3.10.1. Polymorphic sites were called and filtered using SAMtools|BCFtool and manually revised using Integrative Genomics Viewer*
Experimental features	*Data include chloroplast genomes and the polymorphic sites in the chloroplast genomes of the sunflower isonuclear (alloplasmic) CMS lines with different cytoplasmic backgrounds*
Data source location	*Southern Federal University, Rostov-on-Don, Russia*
Data accessibility	*Data are published with this article. The raw reads are available in the FIGSHARE repository at the following link*https://doi.org/10.6084/m9.figshare.7520183*. Complete chloroplast genome is available in GenBank NCBI under accession numbers: MK341448.1, MK341449.1, MK341450.1, MK341451.1 and MK341452.1*
Related research article	*M.S. Makarenko, I.V. Kornienko, K.V. Azarin, A.V. Usatov, M.D. Logacheva, N.V. Markin, V.A. Gavrilova, Mitochondrial genomes organization in alloplasmic lines of sunflower (Helianthus annuus* L.*) with various types of cytoplasmic male sterility, PeerJ. 7 (2018) e5266*

**Table:** 

**Value of the data** • The reported data on the chloroplast DNA polymorphisms in alloplasmic sunflower lines with the same nuclear genome and various cytoplasms are a source for further evolutionary studies and nuclear-cytoplasmic interaction studies. • Chloroplast DNA polymorphism data allows to select an appropriate loci for DNA barcoding • A comparison of polymorphic sites in the sunflower cpDNA can be used to determine variable regions in other taxa. • Polymorphic sites (e.g., single nucleotide polymorphism (SNP) or insertion/deletion (INDEL)) can be used to identify cytoplasmic male sterility (CMS) sources in sunflower and to select CMS for use in breeding.

## 1 Data

Raw sequence reads have been deposited in the FIGSHARE database (https://doi.org/10.6084/m9.figshare.7520183) and assembled chloroplast genomes for five alloplasmic CMS lines have been deposited in GenBank NCBI (MK341448.1, MK341449.1, MK341450.1, MK341451.1 and MK341452.1). Data presented in the text include tables and figures giving information on gene content and variability in these 5 alloplasmic lines of sunflower.

## 2 Experimental design, materials, and methods

### 2.1 Plant material and isolation of cpDNA samples

The plant materials were the sunflower (*Helianthus annuus*) fertile line HA89 and its alloplasmic male sterility analog lines derived on the basis of annual (PET1, PET2 - *H. petiolaris*, ANN2 - *H. annuus*) and perennial (MAX1 - *H. maximilliani*) species of the genus *Helianthus* L. HA89 (PI 599773) is an oilseed inbred line obtained by selection from the high oil content sunflower variety VNIIMK 8931 (Russia, 1949) at the Texas Agricultural Experiment Station (USDA) in 1971. The sunflower alloplasmic CMS lines were taken from the genetic collection of the N. I. Vavilov Institute of Plant Genetic Resources (VIR, Saint-Petersburg, Russia).

Chloroplast fractions were isolated from 14-day sunflower seedlings according to the method of Triboush et al. [1] with our modifications [2]. Briefly, 1 g of leaves from seven plants for each line was selected. Then, 1 g of leaf tissue was homogenized in 10 ml STE buffer (0,4M sucrose, 50 mM Tris pH 7.8, 2 mM EDTA-Na2, 0.2% bovine serum albumin, 0.2% β-mercaptoethanol) and centrifuged. After a series of increasing centrifugal force cycles (500g, 1000g, 3000g and 10000g), a pellet containing chloroplasts was used to extract DNA.

DNA was extracted by PhytoSorb kit (Syntol, Russia), according to the manufacture's instruction. The concentration of the isolated DNA (1 μl DNA, 200μl QuantiFluor dsDNA Dye, 79,8 μl 1X TE buffer) was measured using the fluorometer QuantiFluor ST (Promega, USA).

### 2.2 Library preparation and sequencing

For the preparation of libraries, equally pooled DNA from 7 plants of each sunflower line was used. The next generation sequencing (NGS) libraries preparations were made using 1 ng of DNA and Nextera XT DNA Library Prep Kit (Illumina, Mountain View, CA, USA). The library preparation included standard stages of DNA tagmentation and amplification, following the sample preparation protocol by Illumina. The DNA libraries were cleaned using AMPure XP magnetic beads (Beckman Coulter, USA). The quality of the obtained libraries was checked by capillary electrophoresis on Bioanalyzer 2100 (Agilent, USA). Qualitative analysis showed that in the prepared libraries the adapter dimers are insignificant or completely absent. The average size of NGS libraries distribution was between 400 and 1000 bp. DNA quantitation in NGS libraries was determined using the Qubit fluorometer (Invitrogen, USA) and qPCR (Rotor-Gene 6000, Corbett Research, Australia) [3]. Then, libraries for sequencing were diluted up to the concentration of 8 pM. Fertile line, PET1 and MAX1 DNA libraries were sequenced with 2 × 150 bp on NextSeq 500 platform using High Output v2 kit (Illumina, USA). PET2 and ANN2 DNA libraries were sequenced 2 × 250 bp and 2 × 125 bp on MiSeq and HiSeq2000 platform using MiSeq Reagent Kit v2 and TruSeq SBS Kit v3-HS (Illumina, USA). About 7-14 GB of raw reads were obtained for each sunflower line.

### 2.3 Chloroplast genome assembly

Quality control of the raw reads was done using FastQC [4]. Based on FastQC report the trimming of low quality sequences (quality score below 25; Q25) as well as adapter-derived was performed with Trimmomatic software [5]. After trimming and filtering, sequence reads were assembled with SPAdes Genome Assembler v 3.10.1 using 95 K-mer value and read coverage cutoff value equal to 30.0 [6]. Genome assembly validation was performed using QUAST tool [7] and CONTIGuator tool [8]. Also we used the aligning of the sequence reads to the assembled genome sequence and reference chloroplast genome sequence of *H. annuus* L. (GenBank: NC_007977.1) using Bowtie2 tool version 2.3.3 [9] and BLAST (https://blast.ncbi.nlm.nih.gov/BlastAlign.cgi) [10].

### 2.4 Gene annotation and variability in the chloroplast genome sequences

The programs GeSeq [11] and BLAST (https://blast.ncbi.nlm.nih.gov/BlastAlign.cgi) [10] were used to annotate the assembled genomes. For display of graphical genome map, the OGDRAW tool was used [12].

**Fig. 1:**
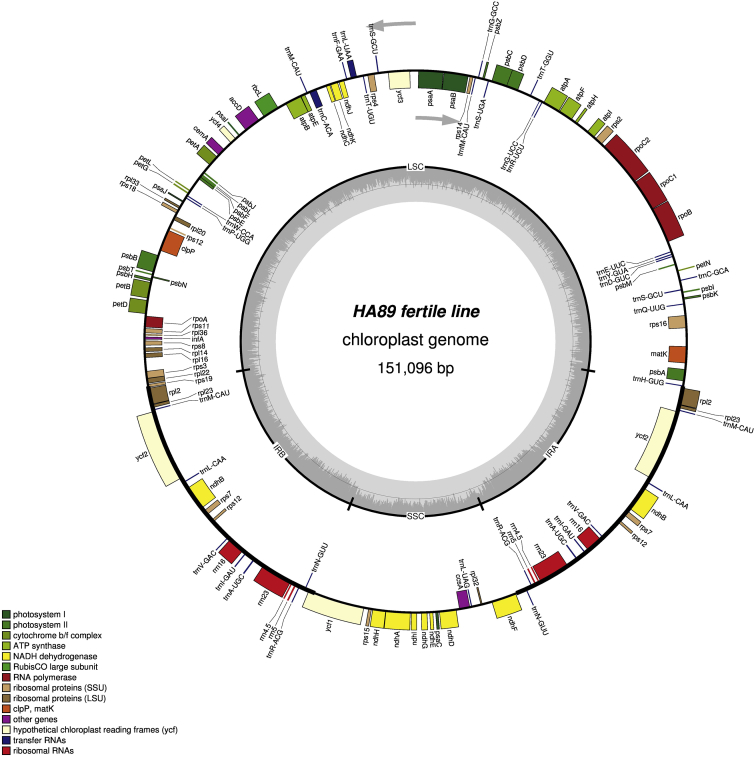
The complete chloroplast genome map of HA89 fertile line. On the outer circle, bold lines indicate the inverted repeats (IRa and IRb). The inner track reflects the GC-content (a dark gray area) and AT-content (a light gray area). Genes annotated outside the circle are transcribed counterclockwise, while those inside are transcribed clockwise. LSC – large single copy region, SSC – small single copy region, IR – inverted repeats.

**Fig. 2:**
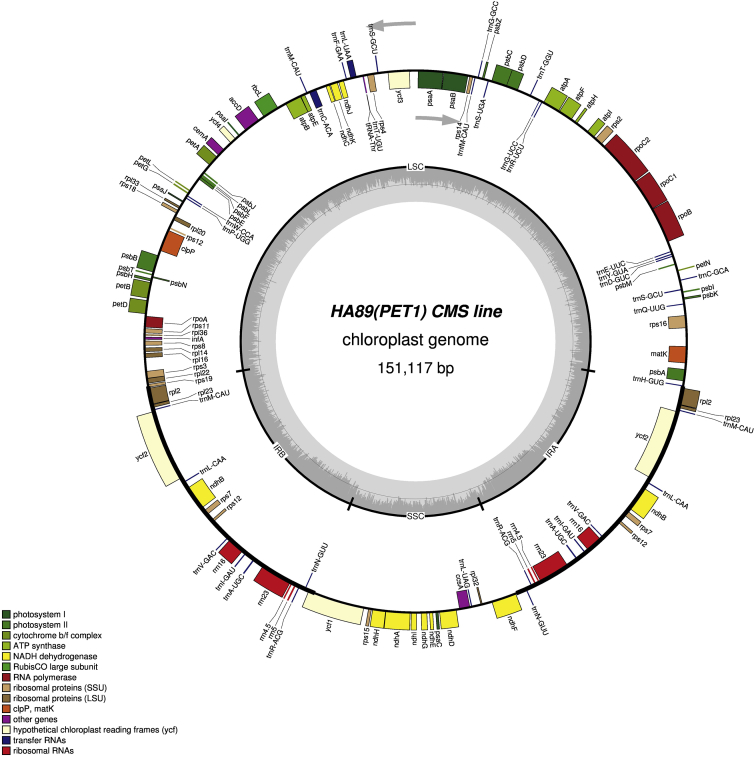
The complete chloroplast genome map of PET1 line. On the outer circle, bold lines indicate the inverted repeats (IRa and IRb). The inner track reflects the GC-content (a dark gray area) and AT-content (a light gray area). Genes annotated outside the circle are transcribed counterclockwise, while those inside are transcribed clockwise. LSC – large single copy region, SSC – small single copy region, IR – inverted repeats.

**Fig. 3:**
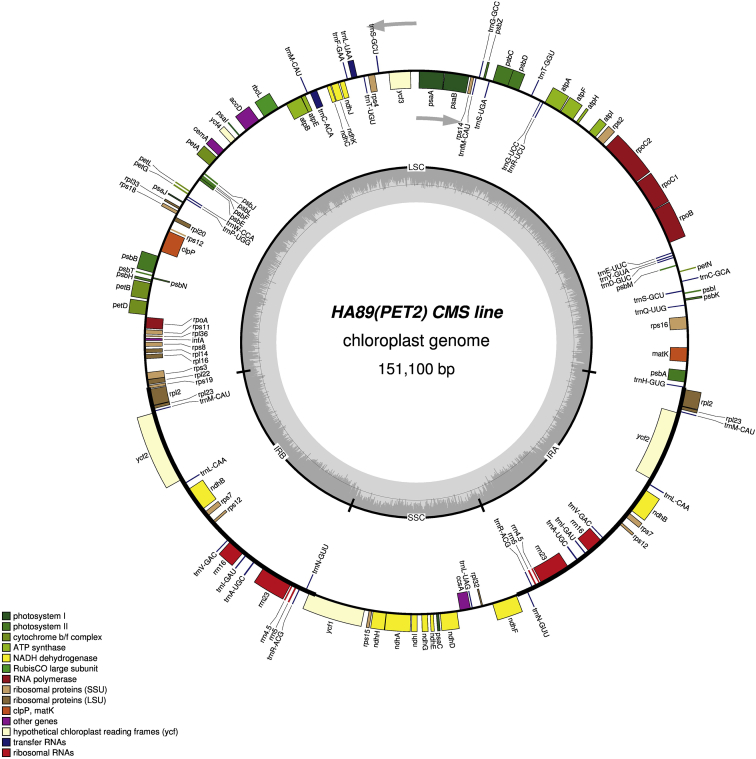
The complete chloroplast genome map of PET2 line. On the outer circle, bold lines indicate the inverted repeats (IRa and IRb). The inner track reflects the GC-content (a dark gray area) and AT-content (a light gray area). Genes annotated outside the circle are transcribed counterclockwise, while those inside are transcribed clockwise. LSC – large single copy region, SSC – small single copy region, IR – inverted repeats.

**Fig. 4:**
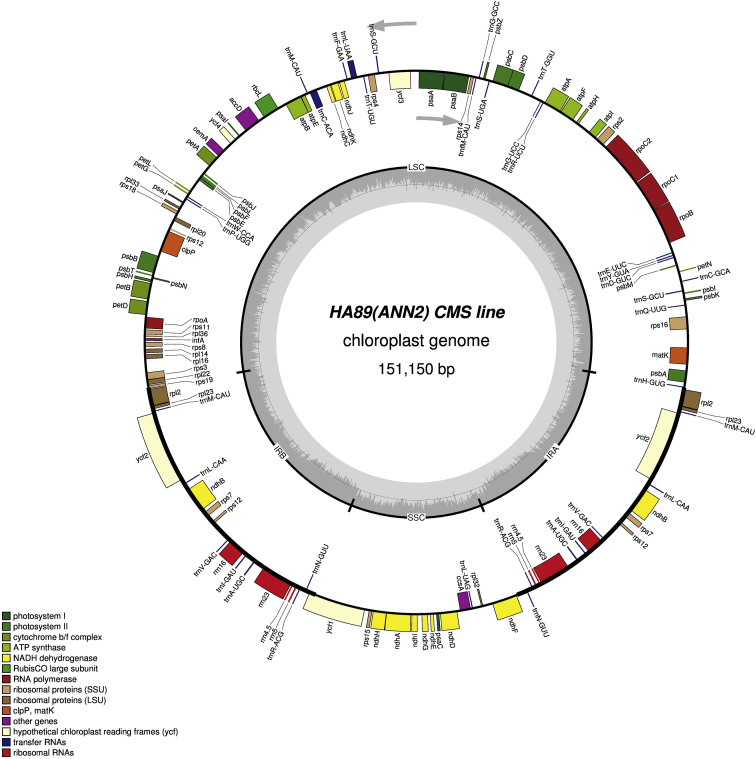
The complete chloroplast genome map of ANN2 line. On the outer circle, bold lines indicate the inverted repeats (IRa and IRb). The inner track reflects the GC-content (a dark gray area) and AT-content (a light gray area). Genes annotated outside the circle are transcribed counterclockwise, while those inside are transcribed clockwise. LSC – large single copy region, SSC – small single copy region, IR – inverted repeats.

**Fig. 5:**
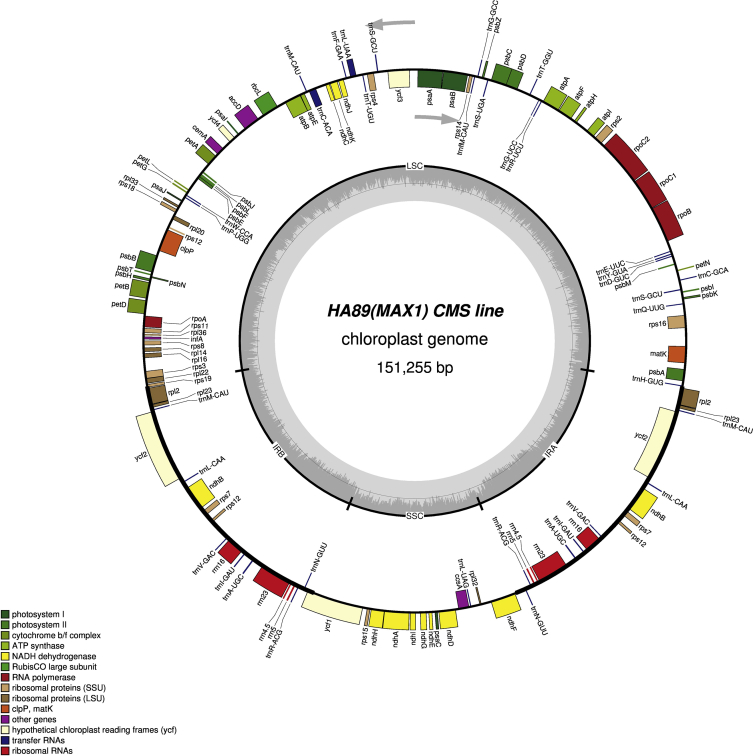
The complete chloroplast genome map of MAX1 line. On the outer circle, bold lines indicate the inverted repeats (IRa and IRb). The inner track reflects the GC-content (a dark gray area) and AT-content (a light gray area). Genes annotated outside the circle are transcribed counterclockwise, while those inside are transcribed clockwise. LSC – large single copy region, SSC – small single copy region, IR – inverted repeats.

It was found that the sizes of the complete chloroplast genome were 151,096 bp (GC: 37.62%) in НА89 fertile line, 151,117 bp (GC: 37.61%) in PET1, 151,100 bp (GC: 37.61%) in PET2, 151,150 bp (GC: 37.61%) in ANN2 and 151,255 bp (GC: 37.58%) in MAX1. The chloroplast genomes has a conservative structure consisting of large single copy region (LSC; ranged from 83,526 bp to 83,711 bp) and small single copy region (SSC; ranged from 18,276 bp to 18,324 bp) separated by a pair of inverted repeats (IR; ranged from 24,610 bp to 24,631 bp). A total of 141 genes were identified, including 90 protein-coding genes, 43 transfer RNA genes, and 8 ribosomal RNA genes (Fig. 1, Fig. 2, Fig. 3, Fig. 4, Fig. 5). Some of them were represented by two or more copies, for example, *trnA*, *rrn23*, etc. Polymorphic sites such as SNP, SSR, insertion and deletion in the chloroplast genome of the studied alloplasmic sunflower lines were detected by alignment against the reference cpDNA sequence (NC_007977.1). Variable sites were called with Sequence Alignment/Map tools (SAMtools)/binary call format tools (BCFtools) package [13] and manually revised using the Integrative Genomics Viewer (IGV) tool [14]. It was identified a total of 472 variable sites, including 314 single-nucleotide polymorphisms, 71 microsatellite polymorphisms and 86 microindels. Detailed data on the polymorphic sites in the chloroplast genomes of the sunflower alloplasmic CMS lines, including a type of variant, position in the reference genome, localization can be found in the Supplementary material. The brief data are summarized in Table 1.

**Table 1:** Number of variable sites identified between the complete chloroplast genome sequences of the reference sequence (NC_007977.1) and HA89 alloplasmic lines (fertile line and four CMS lines).

Line	Type of sequence										
	Genic		Intronic			Intergenic			Total		
	SNP	INDEL	SNP	INDEL	SSR	SNP	INDEL	SSR	SNP	INDEL	SSR
Fertile line HA89	0	1	0	0	2	3	2	3	3	3	5
PET1	9	1	4	0	5	12	5	22	25	6	27
PET2	55	2	13	5	9	89	35	40	158	42	49
ANN2	53	1	10	4	9	82	33	39	145	38	48
MAX1	72	2	11	2	6	110	42	47	193	47	53
