# Supplementary material for: Data on the polymorphic sites in the chloroplast genomes of the sunflower alloplasmic CMS lines
Source: Data Brief. 2019 May 26;25:104072. doi: 10.1016/j.dib.2019.104072 (PMC6562223; doi:10.1016/j.dib.2019.104072)
Supplement: Multimedia component 1 [file mmc1.pdf]

Conflicts of Interest Statement

Manuscript title: Data on the polymorphic sites in the chloroplast genomes of the sunflower alloplasmic CMS lines

We wish to confirm that there are no known conflicts of interest associated with this publication.

We confirm that the manuscript has been read and approved by all named authors and that there are no other persons who satisfied the criteria for authorship but are not listed. We further confirm that the order of authors listed in the manuscript has been approved by all of us.

We understand that the Corresponding Author is the sole contact for the Editorial process (including Editorial Manager and direct communications with the office). We confirm that we have provided a current, correct email address which is accessible by the Corresponding Author and which has been configured to accept email from azkir@rambler.ru

Signed by all authors as follows:
Kirill Azarin (azkir@rambler.ru)
Maksim Makarenko (mcmakarenko@yandex.ru)
Alexander Usatov (usatova@mail.ru)
Oleg Gorbachenko (oleg_gorbachenko@mail.ru)
Alexey Kovalevich (nightmare9606@gmail.com)
Vera Gavrilova (v.gavrilova@vir.nw.ru)
